# Effect of flow on targeting and penetration of angiopep-decorated nanoparticles in a microfluidic model blood-brain barrier

**DOI:** 10.1371/journal.pone.0205158

**Published:** 2018-10-09

**Authors:** Iason Papademetriou, Else Vedula, Joseph Charest, Tyrone Porter

**Affiliations:** 1 Department of Mechanical Engineering, Boston University, Boston, Massachusetts, United States of America; 2 Human Organ Systems, Draper Laboratory, Cambridge, Massachusetts, United States of America; 3 Department of Biomedical Engineering, Boston University, Boston, Massachusetts, United States of America; Hungarian Academy of Sciences, HUNGARY

## Abstract

The blood-brain barrier (BBB) limits transport of nanoparticles from the circulation to the brain parenchyma. Angiopep-2, a peptide which functions as a brain transport vector, can be coupled to nanoparticles in order to facilitate binding and internalization by brain endothelial cells (ECs), and subsequent BBB penetration. This multi-step process may be affected by blood flow over brain ECs, as flow influences endothelial cell phenotype as well as interactions of nanoparticles with ECs. In the present study a microfluidic BBB model was constructed to evaluate binding and internalization by brain ECs, as well as BBB penetration of Angiopep-2 coupled liposomes (Ang2-Liposomes) in static and flow conditions. Ang2 conjugation to liposomes markedly improved binding relative to unconjugated liposomes. Ang2-Liposomes bound and were internalized efficiently by brain endothelial cells after static incubation or with 1 dyne/cm^2^ of fluid shear stress (FSS), while binding was reduced at a FSS of 6 dyne/cm^2^. Penetration of the model microfluidic BBB by Ang2-Liposomes was higher at a FSS of 1 dyne/cm^2^ and 6 dyne/cm^2^ than with static incubation. Analysis of barrier function and control experiments for receptor-mediated penetration provided insight into the magnitude of transcellular versus paracellular transport at each tested FSS. Overall, the results demonstrate that flow impacted the binding and BBB penetration of Ang2-functionalized nanoparticles. This highlights the relevance of the local flow environment for *in vitro* modeling of the performance of nanoparticles functionalized with BBB penetrating ligands.

## Introduction

The blood-brain barrier (BBB) is a unique interface in the brain that tightly regulates the flow of ions, nutrients, and metabolites between flowing blood and brain tissue [1–6]. Brain endothelial cells (ECs) form the primary physical barrier to mass transport across the BBB due to the presence of intercellular tight junction complexes that restrict paracellular transport. Transcellular transport is restricted by limited pinocytosis, and regulated by membrane transporters and receptors that selectively transport substances across brain ECs [7, 8]. While the BBB plays a significant role in maintaining homeostasis and protecting the brain from pathogens and foreign substances, it also hinders the delivery of substances that will benefit a patient. Suboptimal brain penetration is a major obstacle for the development of drugs to treat brain disorders (e.g. cancer, neurodegenerative disorders) [1, 2, 9]. While small, lipophilic molecules can penetrate the BBB via passive diffusion, the majority of drugs, particularly biomolecules (e.g. therapeutic proteins, nucleic acids) are restricted. A number of strategies have been investigated to enable brain delivery, such as administration routes which bypass the BBB (e.g. intranasal, intrathecal), methods to modulate BBB permeability (e.g. osmotic agents, focused ultrasound, tight junction modulating agents), direct biochemical modification of drugs (e.g. to impart lipophilicity), and enabling targeting and transport across the BBB via carrier-mediated transport or receptor-mediated transport [1, 2, 9, 10]. In the case of receptor-mediated transport, a ligand (e.g. antibody, peptide) that enables binding to a receptor (e.g. transferrin receptor, LRP1) present on the luminal surface of brain ECs is either coupled directly to drugs or added to the exterior of drug-loaded nanoparticles [9, 10]. Subsequently, internalization and transcytosis facilitates BBB penetration.

Angiopep-2 (Ang2) is a peptide ligand of LRP1 which enables BBB penetration. Ang2 was derived from the Kunitz protease inhibitor domain, an amino acid sequence found in aprotinin and other natural ligands of LRP1 [11, 12]. Studies of Ang2 BBB penetration using *in vitro* BBB models and *in vivo* established Ang2 as a promising BBB penetrating ligand [11, 12]. Ang2-drug conjugates have reached clinical trials, [10], and Ang2-functionalized nanoparticles are under preclinical development to facilitate delivery of drugs to the brain [13–23]. Several previous studies demonstrated that Ang2-functionalization enhanced BBB penetration of nanoparticles (e.g. gold and polymeric nanoparticles, dendrimers, liposomes, carbon nanodots and nanotubes), providing proof of concept and preclinical validations of this approach [13–20, 22]. Further rational design of this approach depends on improved understanding of how (patho)physiological conditions affect binding and BBB penetration of Ang2-functionalized nanoparticles, so that nanoparticle characteristics (e.g. size, avidity) can be selected to optimize performance.

Luminal fluid flow present in brain capillaries may alter interactions that facilitate binding of Ang2-functionalized nanoparticles with brain ECs. Physiological flow in brain capillaries can vary from approximately 5–23 dyne/cm^2^, [24], while flow can be reduced/static in pathological conditions (e.g. ischemic stroke, cancer, etc). Binding of ligand-functionalized nanoparticles to ECs in the presence of flow has been studied extensively [25–34]. Binding can be augmented by flow due to increased nanoparticle collisions with ECs or by rolling of nanoparticles along the EC surface [35]. On the other hand, flow over ECs imparts fluid shear stress (FSS) which works against nanoparticle avidity [32, 36], and flow with red blood cells can reduce nanoparticle margination [28, 37]. Overall, binding of ligand-functionalized nanoparticles in blood flow depends on a number of interdependent factors including nanoparticle characteristics (e.g. size, avidity) and hemodynamics (e.g. fluid shear rate, blood composition) [28, 29, 35, 38–40]. Binding of Ang2-functionalized nanoparticles has been investigated in static cultures of brain ECs [15, 17, 41], but binding to brain ECs in the presence of flow has not been examined previously.

Luminal fluid flow also induces many changes in endothelial cell phenotype which can modulate penetration of solutes or nanoparticles. Most well characterized is the effect of sustained laminar flow at physiological levels of FSS, the key term here being sustained. Several studies support that sustained flow increases formation of intact tight junction complexes [42–45] and reduces cell turnover (e.g. apoptosis/mitosis), [45–48], both of which can reduce accessibility of the paracellular route. On the other hand, acute exposure to flow or pathophysiologically low level of FSS can stimulate disassembly of tight or adherens junctions, [49–51], increase hydraulic conductivity, [49, 50, 52], and enhance cell turnover, [45, 53], potentially opening the paracellular route to solutes (e.g. dextran, [44], ldl, [54], albumin [55]) or perhaps even nanoparticles. Flow may also alter EC phenotype in ways that promote BBB penetration via receptor-mediated transcytosis. Binding to brain ECs (the first step of receptor-mediated transcytosis) can be enhanced by FSS [56–59]. This is because FSS can increase expression of cell membrane-bound receptors (e.g. cell adhesion molecules, [56, 57], low density lipoproteins (ldl), [58, 59]) that can be targeted by ligand-functionalized nanoparticles [32, 60]. Internalization of nanoparticles by ECs can also be affected by flow-induced changes to EC phenotype. While adaption of ECs to chronic flow reduces internalization of polymer nanoparticles targeted to PECAM-1 or ICAM-1 [30, 61], acute exposure to flow can enhance internalization of PECAM-1-targeted nanoparticles [30, 62]. Internalization of solutes by ECs (e.g. ldl, acetylated ldl, horseradish peroxidase) is also modulated by flow [54, 63, 64]. In addition, FSS increases the density of caveolae, a mechanosensitive domain which mediates transcytosis, on the luminal plasma membrane of ECs [65–67]. Overall, these studies suggest that flow may increase or decrease BBB penetration of Ang2-functionalized nanoparticles, depending upon the magnitude and exposure time of the generated shear stress. BBB penetration of Ang2-functionalized nanoparticles has been observed in static *in vitro* BBB models as well as *in vivo* where sustained flow is present, but the effect of acute flow has not been previously investigated [13, 15–17, 20–23].

To advance our understanding of this phenomenon, a microfluidic BBB model was constructed. Microfluidic models are increasingly utilized for preclinical drug development with the aim of improving the attrition rate of drugs from the laboratory to clinical trials [68, 69]. These systems provide microscale control of fluid flows, tissue architecture, biochemical stimuli (e.g growth factors, cytokines), and biomechanical stimuli (e.g. fluid shear stress, cyclic stretch) in order to simulate the tissue microenvironment with high spatial and temporal precision [68, 69]. Simulating the tissue microenvironment may be particularly relevant for *in vitro* BBB modeling. The microenvironment, moreso than intrinsic characteristics of brain ECs, is thought to be critical to promoting a BBB phenotype [5, 7, 70]. Current microfluidic BBB models do not fully replicate conditions *in vivo*, but are powerful tools which can simulate many aspects of the BBB microenvironment in physiological or pathophysiological conditions [71]. Microfluidic BBB models have simulated luminal FSS [72–77], interstitial fluid flow [78], cyclic strain [78, 79], 3D extracellular matrix [74, 80, 81], capillary-like geometry [80, 82, 83], co-cultures with other cell types of the neurovascular unit [73–77, 81, 84], and optimization of fluid-to-tissue ratio [74, 85]. Microfluidic models have been utilized to investigate hemodynamic effects on binding of nanoparticles [31, 37, 39, 40, 86]. Compartmentalized microfluidic BBB models incorporating flow can be utilized to evaluate BBB penetration and monitor barrier phenotype with fluorescence microscopy [72, 73, 75, 77, 79, 83, 85, 87, 88].

In the present study, brain ECs were grown in the upper microchannel of a compartmentalized microfluidic device to establish a model BBB. Brain ECs were grown on a topographically-patterned substrate, as flow and topography can work synergistically to influence cell phenotype [89]. Ang2-liposomes were then incubated in the upper microchannel in static fluid or subjected to acute flow in order to test effects on binding to brain ECs and BBB penetration.

## Materials and methods

### Microfluidic device structure and fabrication

The microfluidic device structure consists of S-shaped microchannels where the region of overlap is separated by a porous membrane patterned with 1 micron wide pits as described previously [90]. Briefly, polydimethylsiloxane (PDMS) microchannels were molded from SU-8 wafers and bonded to a polycarbonate membrane using a silicone adhesive, followed by oxygen plasma treatment, bonding to a glass coverslip, sterilization with 70% ethanol and autoclave, and washes with phosphate buffered saline (PBS). The upper channel dimensions are 5 mm x 0.5 mm x 0.1 mm in length x width x height, and the lower channel dimensions are 5 mm x 0.25 mm x 0.1 mm (surface area of the region of overlap is 1.25 mm^2^). Fluidic ports were used to introduce liposomes or dextran into the upper channel, and to collect permeated solutes from the lower channel.

### Cell culture

bEnd.3 cells were purchased from American Type Culture Collection in 2015. bEnd.3 cells are a polyoma middle T-transformed mouse brain EC cell line [91]. bEnd.3 cells were cultured in DMEM/F12 with 10% FBS, 1% pencillin/streptomycin, glutamax, 1% β-mercaptoethanol and 15 mM HEPES for 2–3 days, trypsinized, and seeded at a density of 4–8 x 10^4^ cells/cm^2^ in microfluidic devices or cell culture well plates.

For experiments in microfluidic devices, the topographically-patterned polycarbonate membrane was treated with a solution of 100 μg/ml of fibronectin and 100 μg/ml collagen IV in PBS for 15–24 hours at 37°C prior to bEnd.3 cell seeding. bEnd.3 cells were allowed to attach for 2 hrs, and then channels were aspirated to remove unattached cells. The cells were cultured over 3–6 days with daily replacement of the medium prior to experiments.

### Liposome preparation and angiopep conjugation

Fluorescent liposomes were prepared using the lipid film hydration method. Lipids were 1,2-dipalmitoyl-*sn*-glycero-3-phosphocholine (DPPC), 1,2-distearoyl-*sn*-glycero-3-phosphoethanolamine-N [methoxy(polyethylene glycol)-2000 (DSPE-PEG2k), DSPE-PEG maleimide, MW 3400 (DSPE-PEG-MAL3.4k), and 1,2-dipalmitoyl-*sn*-glycero-3-phosphoethanolamine-N-lissamine rhodamine B sulfonyl (DPPE-Rhodamine). A lipid mixture with molar ratio of 91:7:1 DPPC, DSPE-PEG2k, DSPE-PEG-MAL3.4k, and 0.2–1.0 mol% DPPE-Rhodamine were mixed in chloroform and evaporated with an argon gas stream followed by vacuum-desiccation overnight. The lipids were rehydrated in PBS at 55°C for 1–3 hrs, followed by probe sonication for 2 min at 20% power, and sequential extrusion through nucleopore track-etched membranes (Whatman) of sizes 200 nm, 100 nm, and 50 nm with a Lipex 10 ml extruder (Northern Lipids). Angiopep-2, a 19-mer peptide (TFFYGGSRGKRNNFKTEEY), [12], was synthesized and modified with a thiol group at Tufts University Core Facility. Angiopep-2 was added to the prepared liposomes in a 1:1 molar ratio of angiopep-2:DSPE-PEG-MAL3.4k in PBS at pH 7.4. The reaction was incubated for 16–24 hours at room temperature with stirring, followed by removal of unconjugated angiopep-2 via centrifugation with a size-selective filter (Amicon, 10 kDa centrifugal filter). Liposomes were centrifuged at 2400 x g until >95% of the original volume was filtered. PBS or HBSS with calcium and magnesium salts was added to adjust the liposome concentration for experiments. Liposomes with or without Ang2 conjugation as assessed by dynamic light scattering were 80–95 nm in diameter with polydispersity of 0.1–0.3 (effective diameter). A qNANO (Izon Science), which counts nanoparticles based on the Coulter principle, was used to determine liposome concentration in the stock. The stock solutions were then diluted for subsequent experiments based on desired liposome concentration.

### TEER measurement

TEER of confluent monolayers was measured after 4 days culture using Ag/AgCl sintered wire electrodes with 0.20-mm diameter (A-M systems). Electrodes were added to the fluidic ports of the device and connected to an in-house device which adapted the current to a range measurable with an epithelial voltohmeter (EVOM2, WPI). The measured resistance of devices was subtracted from the resistance with no cells present, and multiplied by the surface area of the monolayer (2.5 mm^2^) to calculate TEER.

### Immunofluorescence imaging of tight junction proteins

After exposure to FSS for 2 hours, bEnd.3 monolayers were washed with HBSS containing calcium and magnesium at 37°C to remove medium and cell debris, fixed with 3.7% paraformaldehyde (PFA)/phosphate buffered saline (PBS), washed again to remove excess PFA, blocked for 45 min with 3% BSA/PBS, and permeabilized with 0.3% triton for 10 min. Primary antibody for Claudin-5 (5 µg/ml, rabbit polyclonal, ThermoFisher) was added to the upper channel for 2–3 hours at room temperature. The cells were then washed with PBS, and Alexa-Fluor 488 anti-rabbit IgG was used to label the primary antibody (1:400) for 30 min at room temperature, followed by DAPI for 5 min (40 μg/ml) as a counterstain for cell nuclei.

### Liposome binding and internalization by brain ECs

Liposome binding and internalization was visualized with fluorescence imaging and quantified with fluorescence spectroscopy. For fluorescence imaging experiments, bEnd.3 cells were incubated with Ang2-Liposomes for varying times/concentrations in static fluid or in the presence of FSS (detailed in figure legends), washed to remove unbound liposomes, fixed with 2% PFA, stained with DAPI, imaged with a scanning confocal microscope (Olympus FV1000), and processed using FIJI software. To quantify liposome binding, bEnd.3 cells were grown directly in cell culture plates. Following incubation with Ang2-liposomes, the cells were lysed with 1%Triton/1M NaOH and fluorescence detected with a SpectraMax M5 plate reader (ex/em 560/595 nm) which was converted to liposome concentration using a calibration curve (S1 Fig). For the competition experiment, a rabbit polyclonal anti- mouse LRP1 antibody (MyBioSource) was preincubated with bEnd.3 cells for 45 minutes at 37°C, then co-incubated with Ang2-liposomes for 60 minutes at 4°C.

To assess internalization, Z-stacks of bEnd.3 cells (0.6–0.9 microns per slice) were acquired through the entire cell, and assembled into 3 dimensional images using FIJI software.

### Permeability of dextrans and liposomes in microfluidic devices

Permeability of 4 kDa, 20 kDa, and 500 kDa FITC-dextran (Sigma-Aldrich) and rhodamine-labeled liposomes was assessed by measuring tracer flux across the brain EC cell barrier. Devices were prepared as described in the previous section prior to addition of the tracer. The tracer was then added to the luminal channel (0.05–2 mM FITC-dextran incubated statically for 15–20 min or 120–720 pM liposomes incubated in the presence or absence of flow for 120 minutes). For the competition experiment with antibody for LRP1, anti-LRP was preincubated in devices for 45 min prior to adding Ang2-liposomes with co-incubated anti-LRP (33 μg/ml) for 2 hrs of static incubation. After this time period, a sample from the abluminal channel was collected in 100 μl PBS, and fluorescence intensity was measured using a microplate reader (FITC dextrans: ex/em 488/525 nm, Liposomes: ex/em 560/595 nm). Known masses of FITC-dextran or liposomes were measured under the same excitation/emission settings and sample volume (100 μl PBS) to generate a calibration curve (S1 and S2 Figs), which was used to convert fluorescence signal intensity to mass of dextran or number of liposomes. The permeability coefficient was calculated using the following equation:

$$PS=\frac{\Delta C_{L}V_{U}}{C_{U}A\Delta t}$$ (1)

where ***PS*** is the permeability, ***ΔC***_***L***_ is the change in the lower channel concentration, ***V***_***U***_ is the volume of the upper channel, ***C***_***U***_ is the initial concentration of the upper channel, ***A*** is the membrane surface area, and ***Δt*** is the change in time. The apparent permeability coefficient, ***P***_***app***_, was then calculated as:

$$\frac{1}{P_{app}}=\frac{1}{PS_{cells}}-\frac{1}{PS_{no\;cells}}$$ (2)

where ***PS***_***cells***_ is the permeability across the brain EC barrier and porous membrane, while ***PS***_***no cells***_ is the permeability across the porous membrane alone.

### Statistics

Data were calculated as the mean ± standard error of the mean (SEM). Statistical significance was determined by a Student’s t-test when only two conditions were present, and one-way ANOVA with post hoc Tukey’s test when > 2 conditions were present where p < 0.05 was depicted with one symbol in graphs, p < 0.01 was depicted with two symbols, and p < 0.001 was depicted with 3 symbols.

## Results

**Fig 1:**
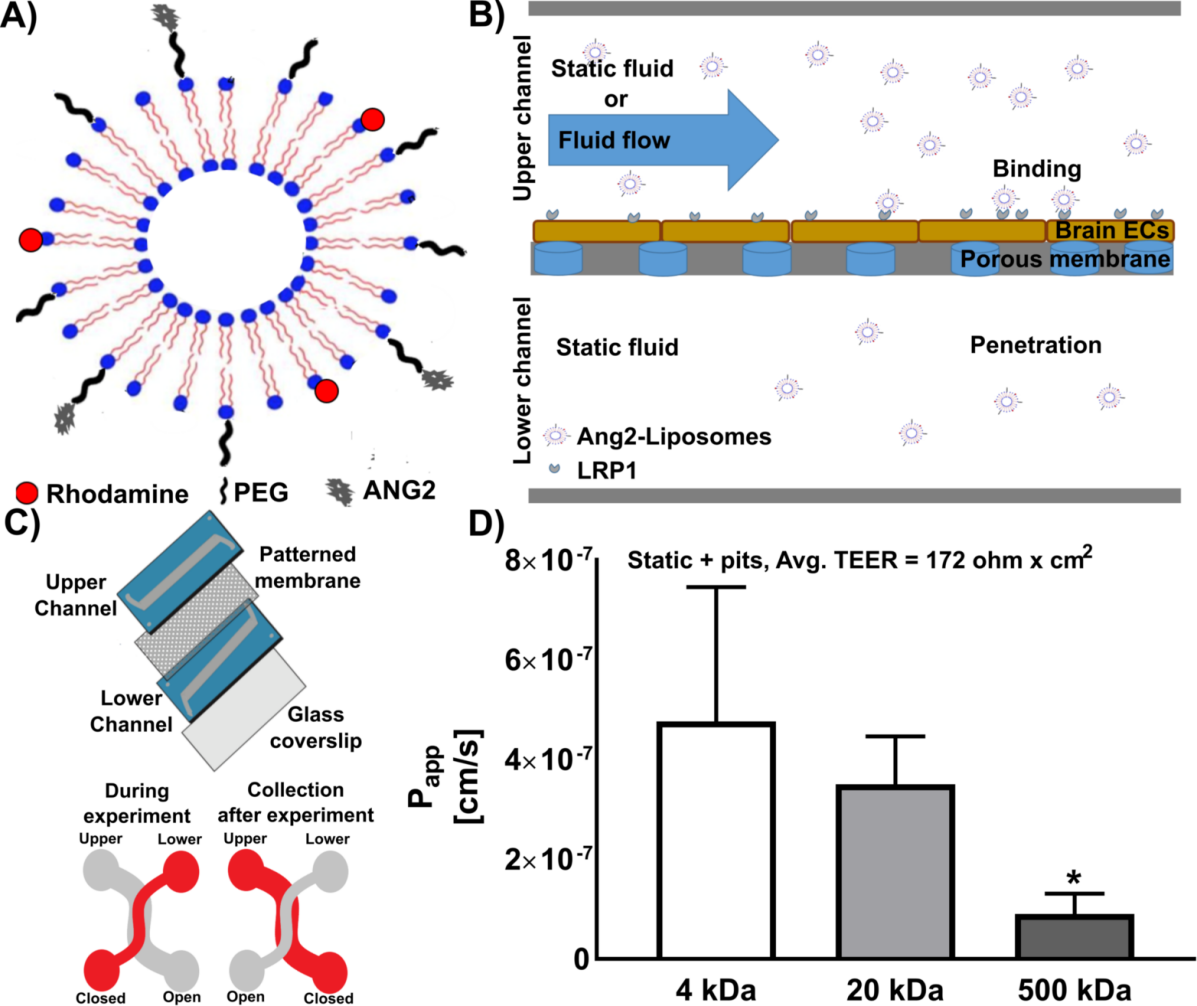
Microfluidic BBB model for evaluation of Ang2-liposome binding and penetration in static or flow condition. A) Cartoon of Ang2-Liposomes. Ang2-Liposomes were fabricated using the lipid film hydration method and sequentially extruded to reach a final size of 80–95 nm. Angiopep-2 was conjugated to the end of PEG chains using maleimide-thiol chemistry. B) Structure of the microfluidic BBB model. Brain ECs (bEnd.3 cells) were grown in the upper channel of devices for 3–6 days to enable barrier formation. Ang2-Liposomes were added to the upper channel and incubated in static fluid or in the presence of flow to assess binding and BBB penetration. Fluid in the lower channel was kept static for the duration of experiments. C) Cartoon depicting layers of the microfluidic BBB model. During the experiment the upper channel was opened to incubate Ang2-Liposomes with flow or in static fluid, while the lower channel was closed to limit advection between the upper and lower channels. After the experiment, the upper channel was closed, and the lower channel was opened to collect samples for analysis. D) After 6 days static culture on pit-patterned membrane, TEER of the microfluidic BBB model reached 172 ohm x cm^2^ (N = 5 devices), and FITC dextran permeability decreased as a function of molecular weight (N ≥ 3 devices per condition). Data are expressed as mean ± SEM.

Liposomes were used as a model nanoparticle for functionalization with Ang2. Ang2-Liposomes were fabricated to a final diameter of 80–95 nm, and contained rhodamine in the lipid shell for detection purposes, PEGylated lipid commonly used to extend circulation time *in vivo*, and Angiopep-2 conjugated to a fraction of the PEGylated lipid (Fig 1A). The microfluidic device structure consisted of two overlapping channels separated by a patterned porous membrane with murine brain ECs (bEnd.3) grown to confluence in the upper channel (Fig 1B and 1C). The device was used to assess binding and internalization of Ang2-Liposomes by brain ECs via fluorescence microscopy, and penetration of the brain EC barrier via fluorescence spectroscopy (Fig 1B). During the experiment the upper channel was opened to incubate Ang2-Liposomes with flow or in static fluid, while the lower channel was closed and kept static to limit advection between the upper and lower channels (Fig 1C). After the experiment the upper channel was closed, and the lower channel was opened to collect samples for analysis.

### Characterization of microfluidic BBB model in static fluid

We first confirmed formation of a functional barrier in the microfluidic BBB model after 3–6 days static culture (i.e. the time frame when experiments of Ang2-Liposome penetration were initiated). Confluent monolayers formed with an average transendothelial electrical resistance (TEER) of 172 ± 8.5 ohm x cm^2^ (Fig 1D). This value correlates with barrier formation in microfluidic BBB models utilizing bEnd.3 cells, whereas baseline TEER is in the range of 30–50 ohm x cm^2^ [72, 83, 92]. An inverse relationship between permeability of solutes and solute size was observed as permeability of FITC-labeled dextrans of sizes 4 kDa, 20 kDa, and 500 kDa was 0.48 x 10^−6^ cm/s, 0.35 x 10^−6^ cm/s, and 0.09 x 10^−6^ cm/s, respectively (Fig 1D), although only the difference between 20 kDa and 500 kDa was statistically significant. Taken together, TEER and the size-selectivity of dextran permeability supported that a functional barrier had formed.

### Binding and internalization of Ang2-liposomes by brain ECs in static fluid or in the presence of flow

**Fig 2:**
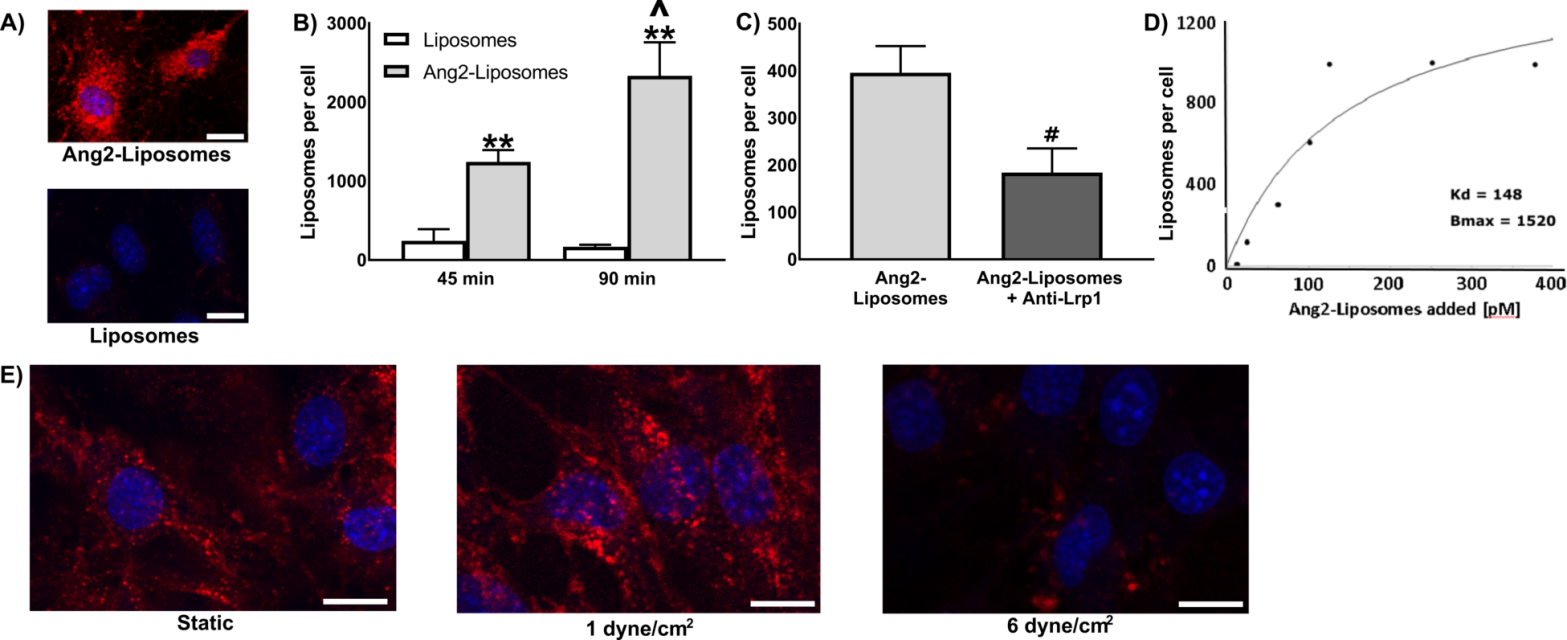
Binding of Ang2-liposomes to brain ECs in static fluid or in the presence of flow. A) Total binding (ie. surface bound + internalized liposomes) of Ang2-liposomes or non-functionalized liposomes in static fluid was visualized by incubation for 90 minutes at 37°C in cell culture plates (N ≥ 2). Cells incubated with non-functionalized liposomes showed negligible fluorescence compared with Ang2-liposomes. Scale bars are ~15 μm. Cell nuclei were labelled with DAPI (blue signal), while liposomes were labelled with rhodamine (red signal). B) Total binding of Ang2-liposomes or non-functionalized liposomes (55 pM) was quantified after 45 or 90 minutes static incubation at 37°C (N ≥ 3). C) Competitive inhibition of Ang2-liposomes binding was performed by pre-incubation with an anti-LRP antibody for 45 minutes at 37°C, then co-incubated with 55 pM Ang2-liposomes (37% of the predicted Kd) for 60 minutes at 4°C (N ≥ 5). D) A saturation binding study at 4°C (i.e. surface binding only) of Ang2-liposomes was performed by static incubation for 60 minutes (N ≥ 2). Following the incubation period in B-D, cells were lysed in 1% Triton/1M NaOH and fluorescence detected in a spectrophotometer. E) Ang2-liposome binding to brain ECs after incubation in static fluid or in the presence of FSS. The concentration of Ang2-Liposomes was ~120 pM in each condition (N = 2). Scale bars are ~10 micron. * compares Liposomes to Ang2-liposomes, ^ compares 45 minutes to 90 minutes, # compares Ang2-liposomes to Ang2-liposomes + anti-LRP1. p < 0.05 was depicted with one symbol in graphs, and p < 0.01 was depicted with two symbols. Data are expressed as mean ± SEM.

We first confirmed the ability of Ang2 to facilitate binding of liposomes to brain ECs in static fluid. Functionalization of liposomes with Ang2 resulted in significant binding to bEnd.3 cells compared with non-functionalized liposomes (Fig 2A and 2B). After incubation for 45 minutes at 37°C (cell binding and internalization occurs concurrently at this temperature), Ang2-liposomes bound bEnd.3 cells at 1244 ± 183 liposomes/cell, an increase of 5.1-fold over binding of non-functionalized liposomes (243 ± 171 liposomes/cell). When incubated for 90 minutes, binding of Ang2-liposomes increased to 2334 ± 489 liposomes/cell, an increase of 13.8-fold over non-functionalized liposomes (169 ± 31 liposomes/cell). Binding of Ang2-liposomes to bEnd.3 cells was inhibited by an antibody recognizing LRP1 which reduced binding of Ang2-liposomes by 59% (Fig 2C), supporting that binding was mediated by interactions between Ang2 and the LRP1 receptor. Binding of Ang2-liposomes was concentration-dependent, saturable, and with avidity in the range of ligand-functionalized nanoparticles [32, 93], providing further evidence that binding of Ang2-Liposomes to brain ECs was mediated by ligand-receptor interactions (Fig 2D).

We next tested the effect of FSS on binding of Ang2-Liposomes. At a concentration of 120 pM Ang2-liposomes for each condition, binding was reduced when incubated at 6 dyne/cm^2^ relative to 1 dyne/cm^2^ or in static fluid. This suggested that while Ang2-Liposomes bound brain ECs efficiently in static fluid or low FSS, nanoparticle detachment forces introduced by FSS overcame the avidity of Ang2-Liposomes at higher FSS.

**Fig 3:**
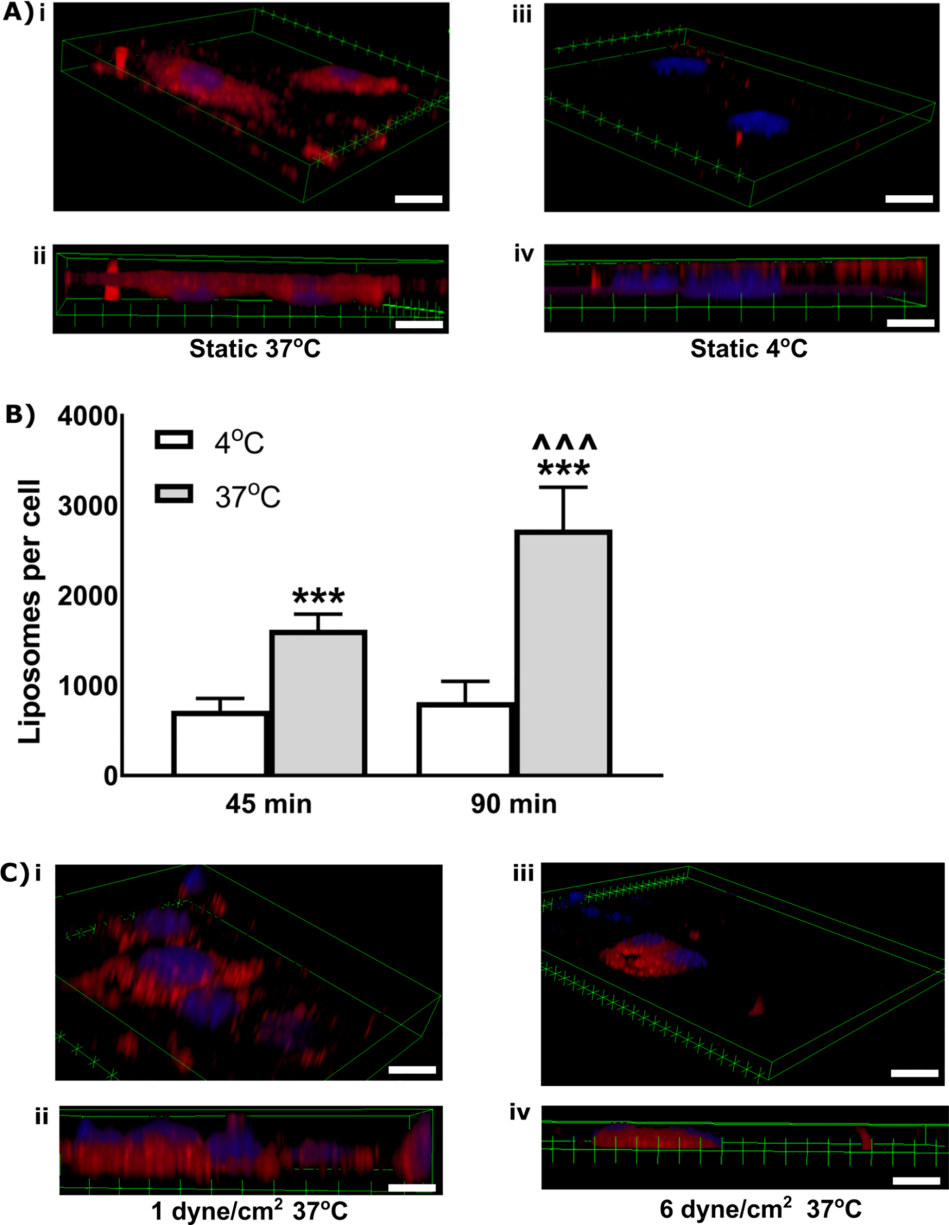
Internalization of Ang2-liposomes by brain ECs in static condition or in presence of flow. A) Internalization of Ang2-liposomes (250 pM) in static fluid after 90 minutes at 37°C vs 4°C. Z-stacks (0.6–0.9 microns/slice) through the entire cell (~6–10 microns) showed perinuclear localization of Ang2-liposomes at 37°C (i) compared with greater localization at the cell periphery at 4°C (iii). A cross-sectional view shows Ang2- liposomes within the cell at 37°C (ii) vs at the cell surface at 4°C (iv) (N ≥ 2). Cell nuclei were labelled with DAPI (blue signal), while liposomes were labelled with rhodamine (red signal). Scale bars are ~10 micron. B) Internalization kinetics and magnitude in static fluid were quantified by comparing binding of Ang2-liposomes at 37°C vs 4°C after 45 and 90 minutes incubation in cell culture plates (N ≥ 3). Following the incubation period, cells were lysed in 1% Triton/1M NaOH and fluorescence detected in a spectrophotometer.* compares 4°C vs 37 ^o^C, and ^ compares 45 minutes vs 90 minutes. The use of 3 symbols for each comparison reflects that p < 0.001. Data are expressed as mean ± SEM. C) Internalization of Ang2-Liposomes (110 pM) in the presence of FSS was assessed after 90 minutes at 37°C. Scale bars are ~10 micron.

Next the internalization of Ang2-liposomes by brain ECs was evaluated by confocal fluorescence microscopy. Z-stacks indicated that Ang2-liposomes were internalized with perinuclear localization observed at 37°C (endocytosis was active, Fig 3A i-ii, whereas Ang2-liposomes were localized mainly at the cell surface at 4°C (endocytosis was inactive, Fig 3A iii-iv). To estimate magnitude and efficiency of internalization in static fluid, binding of Ang2-liposomes over time at 4°C vs 37°C was tested (Fig 3B). Binding of Ang2- liposomes was 2.9-fold greater at 37 ^o^C than at 4 ^o^C after 45-minute incubation (1245 ± 262 liposomes/cell vs. 435 ± 84 liposomes /cell), and 6.9-fold greater after 90-minute incubation (3900 ± 372 liposomes/cell vs. 567 ± 123 liposomes/cell). This suggested that most Ang2-liposomes were internalized by brain ECs rather than remaining on the cell surface, and were internalized relatively rapidly. Having established that Ang2-Liposomes were internalized in static fluid, we next evaluated internalization in the presence of FSS. After 2 hours of incubation at 37°C, internalized Ang2-Liposomes were observed at 1 dyne/cm^2^ and 6 dyne/cm^2^ (Fig 3C). Cells with perinuclear localization of Ang2-Liposomes (e.g. as displayed in Fig 3A and 3C) were selected for analysis via z-stacks. Perinuclear localization was frequently observed at 1 dyne/cm^2^ as in static condition, and was relatively rare at 6 dyne/cm^2^, likely due to reduced binding at 6 dyne/cm^2^. Overall, these studies suggest that FSS significantly impacted binding levels relative to incubation in static fluid, while it remained possible for Ang2-Liposomes to internalize in the presence of FSS.

### Ang-2 liposome penetration of brain ECs in the presence of FSS

**Fig 4:**
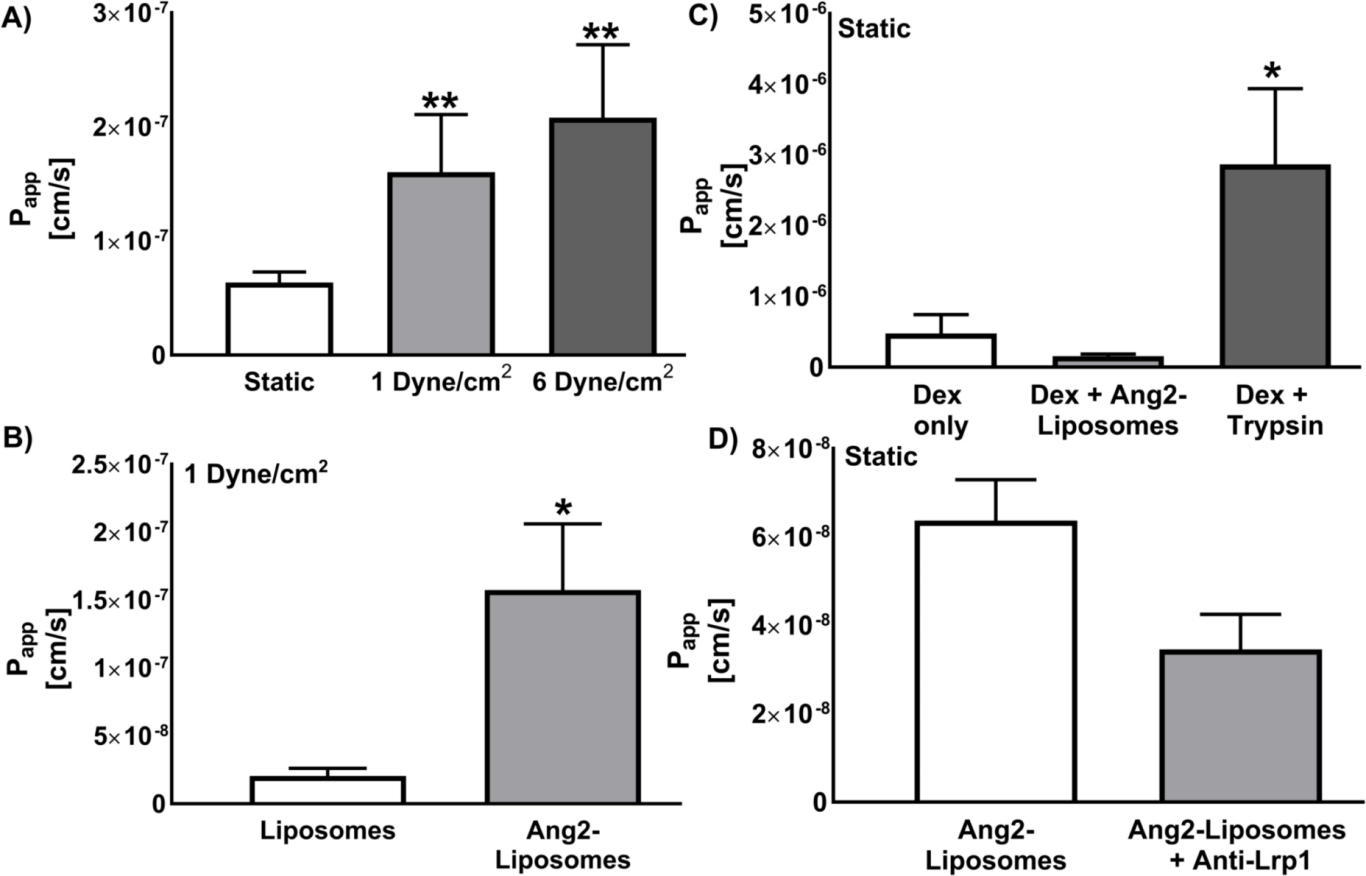
Ang2-liposome penetration of brain ECs in the presence or absence of FSS. A) Penetration of Ang2-liposomes incubated in static fluid or in the presence of FSS was evaluated at 37 ^o^C after 120 minutes incubation. Fluid in the lower channel was kept static during the experiment (N ≥ 4 devices per condition). * compares static to FSS with one symbol where p < 0.05 and two symbols where p < 0.01. B) Penetration of Ang2-functionalized vs. non-functionalized liposomes in the presence of FSS in the upper channel. Fluid in the lower channel was kept static during the experiment (N ≥ 3).C) Induction of barrier disruption by Ang2-liposomes was evaluated by comparing dextran penetration in the presence or absence of Ang2-liposomes at 37°C or trypsin as a positive control (N ≥ 2). Brain EC barriers were incubated with HBSS (Ca^2+^, Mg^2+^) only, Ang2-liposomes, or trypsin for 120 minutes in static fluid in the upper and lower channels. After this period, penetration of 4 kDa dextran was measured after 15–30 minutes static incubation. * compares Dex + Trypsin to Dex only where p < 0.05. D) Competitive inhibition of Ang2-liposome penetration was evaluated with an anti-LRP antibody with static fluid in the upper and lower channels. Anti-LRP was preincubated for 45 minutes followed by co-incubation with Ang2-Liposomes for 120 minutes at 37°C (N ≥ 7). Data are expressed as mean ± SEM.

We next evaluated penetration of the microfluidic BBB model by Ang2-liposomes as a function of FSS. Ang2-liposome penetration was observed in static fluid, and in the presence of FSS (Fig 4A). Penetration of Ang2-liposomes was 1.6±0.6 x 10^−7^ cm/s at 1 dyne/cm^2^ and 2.1±0.7 x 10^−7^ cm/s at 6 dyne/cm^2^, an enhancement of 2.7-fold and 3.5-fold relative to penetration in static fluid (0.6±0.1 x 10^−8^ cm/s). Functionalization of liposomes with Ang2 enhanced penetration by 8-fold at 1 dyne/cm^2^ (1.6±0.5 x 10^−7^ cm/s vs 0.2±0.4 x 10^−7^ cm/s for Ang2-liposomes vs. non-functionalized liposomes, respectively, Fig 4B). Ang2-Liposomes did not appear to affect barrier function, as Ang2-Liposomes co-incubated with dextran in static fluid did not affect dextran permeability, whereas co-incubation of dextran with trypsin significantly enhanced dextran penetration (Fig 4C). Competitive inhibition with anti-LRP1 antibody in static fluid reduced the penetration of Ang2-liposomes by 37%, although this reduction was not statistically significant (p = 0.12, Fig 4D).

**Fig 5:**
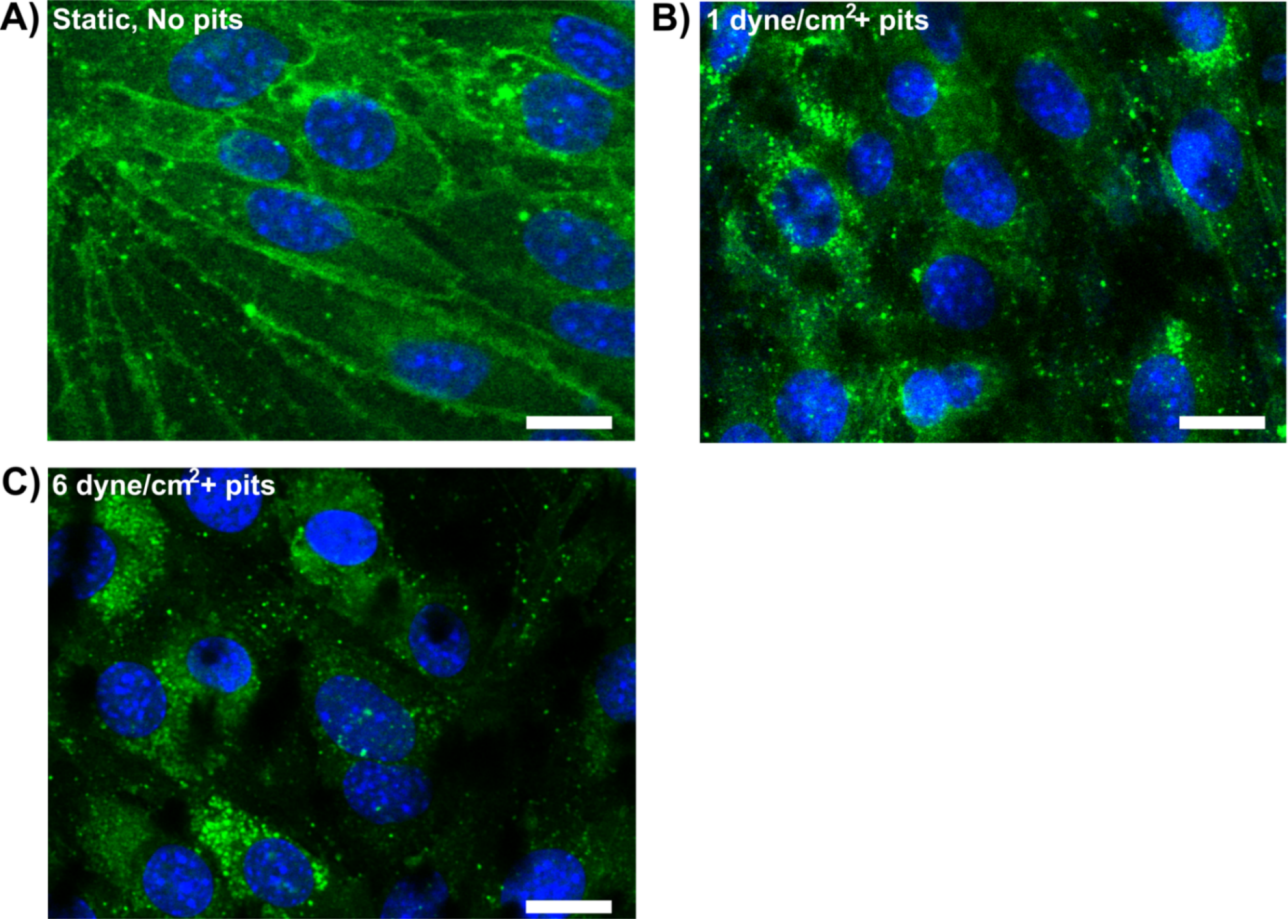
Characterization of microfluidic BBB model function. A) Claudin-5 staining of brain ECs cultured for 6 days in static cell culture plates. B-C) Claudin-5 staining after 6 days static culture followed by 2 hours flow in the upper channel of the microfluidic BBB model (brain ECs cultured on pit-patterned membrane, N = 2 per condition). Scale bars are ~ 10m micron.

A key question of interest was the distribution of tight junction proteins after culture of brain ECs on pit-patterned substrate and acute exposure to FSS. As shown in Fig 5, the expression of Claudin-5 appeared primarily perinuclear in brain EC cultures after 2 hours of flow exposure (i.e. 6 days static incubation of brain ECs on pit-patterned membranes, followed by 2 hours of flow, Fig 5). This was in contrast to expression of Claudin-5 at intercellular borders with brain ECs cultured in cell culture plates (6 days static incubation). The observation suggested that tight junctions were not fully intact during the flow exposure, which may have influenced the penetration of nanoparticles through the model BBB.

## Discussion

Nanoparticles functionalized with Ang2 are under development to improve treatment of diseases affecting the brain [13–22]. Understanding how the BBB microenvironment affects binding and BBB penetration is critical to optimizing performance of Ang2-functionalized nanoparticles in (patho)physiological conditions. Luminal fluid flow over brain ECs is an aspect of the BBB microenvironment which merits investigation in this context, as flow impacts nanoparticle interactions with ECs, and modulates EC phenotype [28–30, 35, 38–40]. In the present study, a microfluidic BBB model was utilized to examine the effect of flow on binding and BBB penetration of Ang2-functionalized liposomes.

Binding of Ang2-Liposomes to brain ECs was efficient in static fluid or at low FSS (1 dyne/cm^2^), but was inhibited at higher FSS of 6 dyne/cm^2^ (Fig 2E). This bears consideration for the design of Ang2-functionalized nanoparticles, as physiological FSS in brain capillaries ranges from approximately 5–23 dyne/cm^2^ [24, 94]. Designing Ang2-nanoparticles with higher avidity via the multivalent effect could help withstand nanoparticle detachment forces imparted by the presence of flow. However, increasing avidity beyond a certain threshold can reduce BBB penetration of ligand-functionalized nanoparticles [14, 95]. It may be necessary to find a balance by designing avidity to enable binding to brain ECs in the presence of FSS, and to maximize subsequent BBB penetration. Other nanocarrier characteristics (e.g. size, shape) can also influence binding and subcellular transport, [96], and may aid in optimizing BBB penetration. On the other hand, lowering avidity of Ang2-nanoparticles could in theory be exploited to preferentially target vasculature where low FSS is present. Relatively few studies have examined the impact of flow on binding of ligand-functionalized nanoparticles <100 nm in size, and this size regime is suited for BBB penetration.

Internalization of Ang2-Liposomes was observed in the presence of FSS (Fig 3). This is consistent with evidence supporting internalization and receptor-mediated transcytosis of Ang2-nanoparticles *in vivo* where flow is present [13, 15–17, 20–23]. Ang2-liposomes were internalized rapidly (i.e. within 45 minutes, most bound Ang2-Liposomes were internalized, Fig 3B). This is consistent with the rapid penetration kinetics observed with Ang2-nanoparticles in BBB models [17, 22]. The magnitude of internalized Ang2-Liposomes increased between 45 and 90 minutes, suggesting that binding and internalization occurred continuously for at least 45 minutes without saturation of the LRP1 receptor pool, or endocytic machinery.

Penetration of the BBB model by Ang2-Liposomes was enhanced in the presence of flow relative to static incubation. At 1 dyne/ cm^2^, the results collectively suggest that penetration of the BBB model was primarily via receptor-mediated transport. This is because binding and internalization of Ang2-Liposomes by brain ECs remained efficient (Fig 2E, Fig 3C), penetration of non-functionalized liposomes was considerably less than Ang2-Liposomes (Fig 4B), and Ang2-Liposomes did not compromise barrier integrity (Fig 4C). Perinuclear localization of Claudin-5 after 2 hours of flow at 1 dyne/cm^2^ suggested that at least partial disassembly of tight junctions had occurred (Fig 5), although the relative contribution of brain EC culture on pit-patterned membrane or exposure to flow was not assessed. BBB integrity is maintained in the absence of Claudin-5 for solutes larger than 800 daltons, [97], which supports that paracellular transport of Ang2-liposomes (80–95 nm in size) may have been restricted at 1 dyne/cm^2^ despite lack of Claudin-5 at cell junctions. In addition, tight junction disassembly is thought to precede disassembly of adherens junctions [45]. Thus, adherens junctions may have remained intact despite perinuclear localization of Claudin-5. It is tempting to speculate that flow-induced upregulation of endocytic signaling enhanced penetration of Ang2-Liposomes via receptor-mediated transcytosis. In support of this, acute FSS of 1–15 dyne/cm^2^ applied to static ECs enhances fluid phase endocytosis of horseradish peroxidase in a magnitude-dependent manner over 2 hours [63]. Additionally, transcytosis of Ang2-functionalized nanoparticles has been reported to occur via caveolae, [41], and acute FSS can increase presence of caveolae at the plasma membrane of ECs [65–67]. However, detailed mechanistic studies of Ang2-liposome penetration are needed to substantiate this hypothesis.

In the case of Ang2-Liposome penetration at 6 dyne/cm^2^, opening of the paracellular route appears more likely. This is because binding to brain ECs was reduced relative to static condition (Fig 2E), while penetration of the BBB model was enhanced (Fig 4A). It is possible that flow at 6 dyne/cm^2^ induced paracellular opening to a greater extent than at 1 dyne/cm^2^. Acute changes in FSS can induce transient disassembly of junction complexes, [49–51], and increase endothelial cell turnover, [45, 53], both of which are characteristic of leaky EC junctions [45]. These changes can be time or magnitude dependent, supporting that FSS of 6 dyne/cm^2^ may have enhanced paracellular transport of Ang2-Liposomes to a greater extent than 1 dyne/cm^2^ in the present study.

A limitation of our study is the use of immortalized brain ECs and lack of co-culture with other cells of the neurovascular unit [92]. bEnd.3 cell monocultures were used in the present study due to their durability, demonstrated barrier function in published microfluidic BBB models, [72, 73, 83], and expression of LRP1 for testing of Ang2-Liposomes [22]. However, co-culture models and models incorporating primary cells better reflect *in vivo* physiology as the phenotype of brain ECs incorporates signaling input from other cells of the NVU [7, 92]. LRP1 expression and accessibility, as well as endocytic signaling could in theory be affected by signaling from astrocytes or pericytes.

## Conclusion

Understanding the role of the BBB microenvironment on the targeting and penetration nanoparticles is critical to developing more effective treatments for diseases affecting the brain. Flow impacts nanoparticle-EC interactions and can alter EC phenotype in a manner which influences binding and BBB penetration of Ang2-functionalized nanoparticles. The present study demonstrated that flow modulates binding to brain ECs and BBB penetration of Ang2-functionalized nanoparticles, highlighting the need for *in vitro* BBB models which replicate the local flow environment. Further investigation of these findings in more physiologically representative *in vitro* models is warranted. Future studies may focus on tuning nanoparticle characteristics (e.g. Ang2 valency) to enable binding in the presence of flow, while maximizing BBB penetration.

## Supporting information

S1 Fig Representative calibration curves of Ang2-liposomes. The concentration of liposomes in the x-axis, expressed as the number of liposomes, in units of picomoles per liter of solution (pM), was determined using a qNANO (Izon Science). Fluorescence in the y-axis was measured using a SpectraMax M5 plate reader where the volume of all samples was kept constant at 100 µl of 1%Triton/1M NaOH (a) or in 100 µl of PBS (b). Background fluorescence of 100 µl of 1%Triton/1M NaOH (a) or PBS (b) with no added liposomes was substracted from each reading.
(DOCX)

S2 Fig Representative calibration curves of fluorescent dextrans. The concentration of dextran in the x-axis, expressed as the number of dextrans, in units of micromoles, per liter of solution (µM), was determined by adding a measured mass of dextran to a measured volume of PBS. Fluorescence in the y-axis was measured using a SpectraMax M5 plate reader where the volume of all samples was kept constant at 100 µl of PBS. Background fluorescence of 100 µl of PBS with no added dextran was substracted from each reading.
(DOCX)
