# Supplementary material for: Effect of flow on targeting and penetration of angiopep-decorated nanoparticles in a microfluidic model blood-brain barrier
Source: PLoS One. 2018 Oct 9;13(10):e0205158. doi: 10.1371/journal.pone.0205158 (PMC6177192; doi:10.1371/journal.pone.0205158)
Supplement: S1 Fig — The concentration of liposomes in the x-axis, expressed as the number of liposomes, in units of picomoles per liter of solution (pM), was determined using a qNANO (Izon Science). Fluorescence in the y-axis was measured using a SpectraMax M5 plate reader where the volume of all samples was kept constant at 100 µl of 1%Triton/1M NaOH (a) or in 100 µl of PBS (b). Background fluorescence of 100 µl of 1%Triton/1M NaOH (a) or PBS (b) with no added liposomes was substracted from each reading. (DOCX) [file pone.0205158.s001.docx]

**Supplemental Fig 1a: A representative calibration curve of Ang2-Liposomes in 1%Triton/1M NaOH.**

**Supplemental Fig 1b: A representative calibration curve of Ang2-Liposomes in PBS.**
