# Supplementary material for: Effect of flow on targeting and penetration of angiopep-decorated nanoparticles in a microfluidic model blood-brain barrier
Source: PLoS One. 2018 Oct 9;13(10):e0205158. doi: 10.1371/journal.pone.0205158 (PMC6177192; doi:10.1371/journal.pone.0205158)
Supplement: Supplementary file 2 [file pone.0205158.s002.docx]

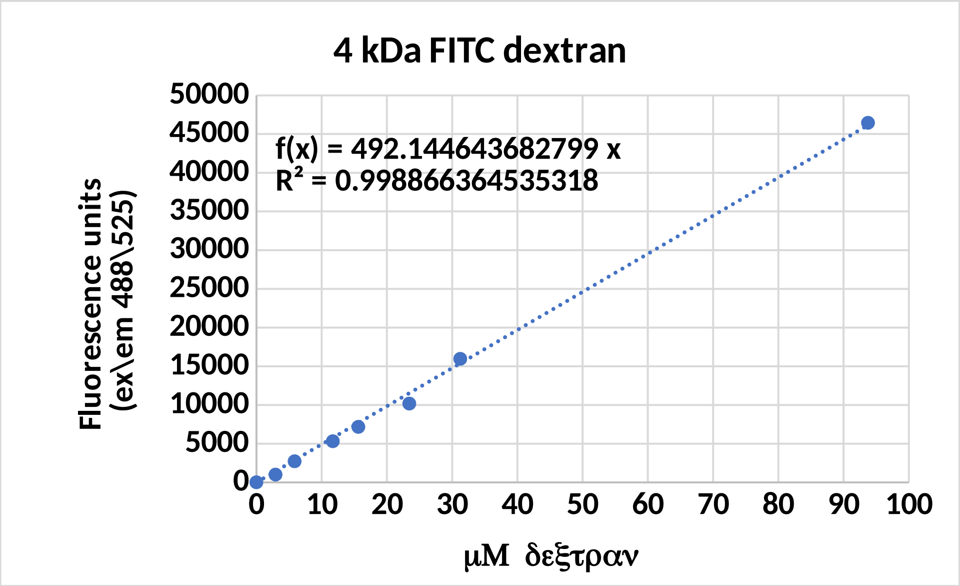

4 kDa FITC dextran
f(x) = 492.144643682799 x
R² = 0.998866364535318
Fluorescence units (ex\em 488\525)
50000
45000
40000
35000
30000
25000
20000
15000
10000
5000
0
0 10 20 30 40 50 60 70 80 90 100
μΜ δεξτραν

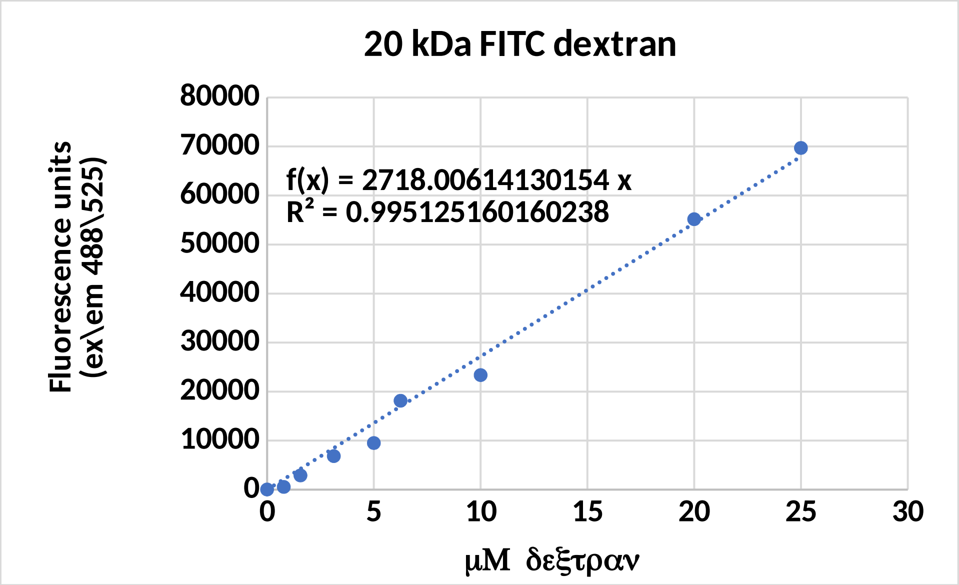

20 kDa FITC dextran
f(x) = 2718.00614130154 x
R² = 0.995125160160238
Fluorescence units (ex\em 488\525)
80000
70000
60000
50000
40000
30000
20000
10000
0
0 5 10 15 20 25 30
μΜ δεξτραν

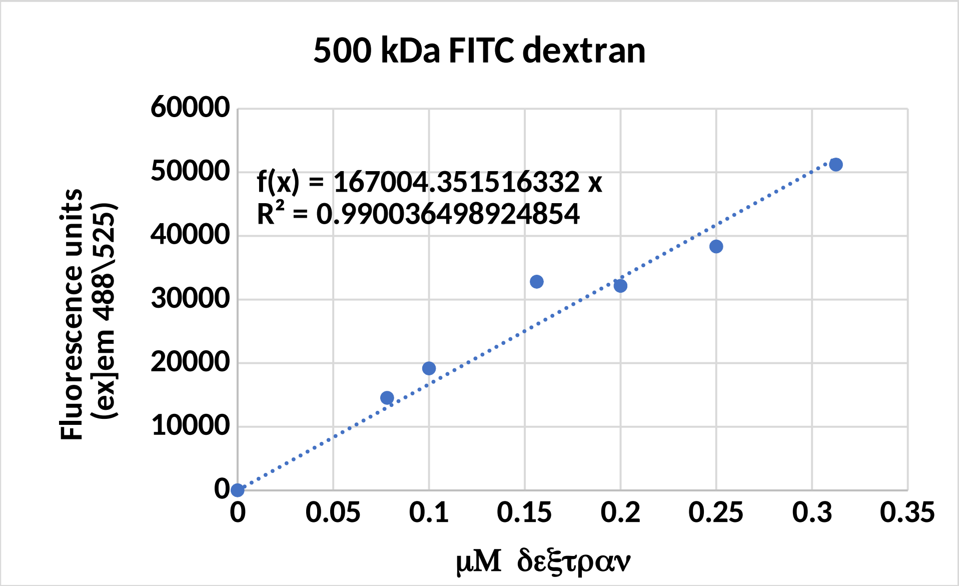
500 kDa FITC dextran
Fluorescence units (ex]em 488\525)
μM δεξτραν
f(x) = 167004.351516332 x
R² = 0.990036498924854
60000
50000
40000
30000
20000
10000
0
0
0.05
0.1
0.15
0.2
0.25
0.3
0.35
